# Flow cytometry protocol for cell death analysis in glioblastoma organoids: A technical note

**DOI:** 10.1371/journal.pone.0327660

**Published:** 2025-09-23

**Authors:** Anna-Laura Potthoff, Meng-Chun Hsieh, Ahmad Melhem, Susanna S. Ng, Barbara E. F. Pregler, Annika Vieregge, Markus Raspe, Lea L. Friker, Thomas Zeyen, Julian P. Layer, Andreas Dolf, Marieta I. Toma, Andreas Waha, Torsten Pietsch, Mike-Andrew Westhoff, Hartmut Vatter, Michael Hölzel, Ulrich Herrlinger, Matthias Schneider

**Affiliations:** 1 Department of Neurosurgery, University Hospital Bonn, Bonn, Germany; 2 Brain Tumor Translational Research Group, University Hospital Bonn, Bonn, Germany; 3 Institute of Neuropathology, University Hospital Bonn, Bonn, Germany; 4 Institute of Experimental Oncology, University Hospital Bonn, Bonn, Germany; 5 Department of Neurooncology, Center for Neurology, University Hospital Bonn, Bonn, Germany; 6 Department of Radiation Oncology, University Hospital Bonn, Bonn, Germany; 7 Flow Cytometry Core Facility, Medical Faculty, University of Bonn, Bonn, Germany; 8 Department of Pathology, University Hospital Bonn, Bonn, Germany; 9 Department of Pediatrics and Adolescent Medicine, University Medical Center Ulm, Ulm, Germany; Bauer Research Foundation, UNITED STATES OF AMERICA

## Abstract

Tumor organoid models have emerged as a promising tool in cancer research. By preserving intra- and intertumoral heterogeneity and structural integrity they provide a physiologically relevant platform for drug-response studies. However, valid methodological approaches for cell death analyses applying flow cytometry, particularly in complex, large organoids, are lacking. Using glioblastoma organoids (GBOs), we developed a flow cytometry protocol to quantify cell death as an important readout in cancer research. Human GBOs were generated out of tumor material from six patients. Temozolomide (TMZ) and lomustine (CCNU) were used as cytotoxic agents commonly employed in glioblastoma therapy. After treatment for 144 and 288 hours, single cell suspensions from densely-packed GBOs were generated through a combined approach of enzymatic and mechanical dissociation. Cells were permeabilized with Triton X and subsequently stained with propidium iodide (PI). PI staining labels fragmented nuclear DNA, yielding a hypodiploid sub-G1 peak in flow cytometry that markes cell death. After treatment for 288 hours with physiologically-relevant concentrations of TMZ and CCNU cell death rates reached up to 63% in our GBO model. Across three GBO populations, the impact of CCNU at the given concentration was more pronounced compared to that observed with TMZ and the cell death rates of treatment for 288 hours surpassed that of the 144-hour treatment. Both biological and technical replicates showed low variability. Hoechst 33258 staining on the same samples confirmed trends in cell death rates obtained from PI-based analysis. We further validated the treatment-induced effect using a plate-based lactate dehydrogenase release assay and measurements of GBO diameter. Our single-stain flow-cytometry protocol scales to large, dense organoids and provides a practical balance of performance, hands-on time, cost, specificity, and throughput. This protocol could support development and evaluation of subtype-specific therapeutic strategies in translational cancer research.

## Introduction

Human organoid models have emerged as a powerful tool in cancer research, offering a more accurate representation of human tumors compared to traditional cell lines [1–3]. Organoids are three-dimensional cultures from patient-derived tumors that mimic the structure and function of the original tissue [4]. In recent years multiple protocols have been developed for the generation of organoids, employing diverse techniques from air-liquid interfaces to mechanical or enzymatic dissociation methods [4–8]. The versatility of organoids is evident from their utilization across a broad spectrum of cancer entities [3], including lung, breast, liver, and intestinal cancers [6,7,9,10] as well as tumors of the central nervous system (CNS), like meningioma or glioblastoma [11–14].

For glioblastoma organoids (GBOs) it has been demonstrated that not only clonal selection of specific subpopulations may be avoided, but even the tumor’s local cytoarchitecture can be preserved [13]. Beyond tumor cells, fresh tumor organoids also contain non-neoplastic cell types such as macrophages, microglia, and endothelial cells [12]. As previously reported, the number of immune cells gradually declines over time, with only a few remaining after approximately eight weeks. However, tumor organoids can be co-cultured with additional cell types including stromal, endothelial, and immune effector cells to further expand their applicability [12,13,15]. Therefore, GBOs facilitate an in-depth exploration of the heterogeneity inherent in cancer cells [16,17], interactions between tumor and stroma [18], and mechanisms driving therapy resistance [19]. Moreover, GBOs offer a physiologically relevant platform to investigate drug responses [3,4,8,14,17,20] and to support development of therapies targeting defined tumor subtypes, cellular subpopulations within tumors, the tumor microenvironment, or malignant tumor networks [21–23].

A pivotal outcome measure in drug testing is cell death [24]. While protocols exist for certain entities utilizing live-dead viability assays with Hoechst, propidium iodide (PI), as well as annexin and calcein, they predominantly rely on fluorescence microscopy for analysis [25,26]. This becomes challenging for several 3D organoid cultures, particularly when considering their size and density. Jacob et al. reported that a single GBO can attain a size of up to 2 mm containing up to almost 1.2 million cells [12]. Comparable sizes and cellular densities have also been reported for organoids from other tumor entities [27,28]. For such dense and large organoids, imaging-based approaches to assess cell death can be suboptimal because dye and light penetration are limiting. Imaging frequently labels only outer layers, which constrains interpretability. If organoids need to be dissociated or dissection for imaging, the advantage of preserved architecture is at least in parts lost while time and cost remain substantial. Plate assays are a good option for high-throughput screening as they are easy to handle and cost-effective. However, in organoids they face two practical challenges: (i) unknown and variable cell numbers per well, to which plate readouts are highly sensitive; and (ii) workflows that assume adherent 2D monolayers with multiple wash steps, which are difficult to implement at scale on dissociated organoid suspensions. Furthermore, plate assays often measure metabolic activity or overall viability/cytotoxicity, which makes them less specific for quantifying cell death.

Given these challenges, flow cytometry emerges as a promising alternative. However, there is a noticeable lack of established protocols customized for organoids. In contrast to well-based plate readouts, our PI-based flow cytometry protocol quantifies cell death at single-cell resolution. By dissociating GBOs and applying debris/doublet gating, we measure hypodiploid (sub-G1) events directly, rather than inferring death from metabolic or protein surrogates. This makes the readout independent of equal pre-seeding across wells which is a key advantage when organoid size and cellularity vary and robust in large, dense organoids. The assay is sensitive to DNA fragmentation (a direct death endpoint) and can simultaneously provide DNA-content cell-cycle information if wanted.

PI is frequently employed as fluorochrome in flow cytometry, facilitating the evaluation of cellular DNA content [29–31]. In this study, we present a detailed protocol for measuring cell death of PI-stained nuclei in GBOs using flow cytometric analysis. Addressing the dense nature of GBOs, our approach combined both enzymatic and mechanical dissociation to achieve a thorough single-cell suspension prior to staining without compromising cell viability. Building on the concept introduced by Nicoletti et al. [31,32] of permeabilizing tumor cells with Triton-X followed by subsequent staining with PI, we established a protocol tailored explicitly for complex organoids. In apoptotic cells, DNA undergoes partial degradation, and permeabilization (through Triton-X) leads to the extraction of low-molecular-weight DNA, while the non-degraded DNA remains within the cell nucleus. These cells manifest as hypodiploid cells, identifiable through the sub-G1 peak in flow cytometry [31]. However, it’s essential to emphasize that numerous types of apoptosis exist, and the extensive fragmentation and loss of DNA fragments are not universally observed in apoptotic death [33,34]. Additionally, necrotic cells occasionally exhibit varying degrees of DNA degradation, which may lead to the formation of hypodiploid nuclei. In this context, we quantified hypodiploid cells represented by the sub-G1 peak [32] as a proxy for cell death without specifying the precise type of cell death.

In many applications, PI is combined with Annexin V. While Annexin/PI-based panels are valuable for distinguishing early from late apoptosis and necrosis, our aim here is a robust, scalable quantification of overall cell death in organoids. Our protocol uses a single stain, is cost-efficient, and simplifies analysis (single channel, minimal compensation, straightforward gates), which reduces inter-operator variability. For that purpose, a direct DNA fragmentation endpoint provides the needed specificity with fewer reagents and lower analytical complexity and costs.

We tested our protocol by administering two standard chemotherapeutic agents in glioblastoma treatment, temozolomide (TMZ) and lomustine (CCNU), to GBOs across 144 and 288 hours [35,36]. As a clinical correlation we provide the responses for TMZ stratified by *MGMT* promoter methylation status of the patient-derived GBOs. Obtained results were cross-verified with Hoechst 33258 staining, a plate-based lactate dehydrogenase (LDH) release assay and measurements of GBO diameter.

To the best of our knowledge, this represents the first comprehensive step-by-step protocol to assess cell death responses in complex organoids using flow cytometry. Our single-stain flow cytometry protocol scales to large, dense organoids and provides a practical balance of performance, hands-on time, cost, specificity, and throughput. Furthermore, we provide comparisons against plate-based and imaging-based methods for assessing treatment-induced effects. Although it remains to be determined whether these readouts correlate with individual patients’ clinical outcomes, this protocol could support the development and evaluation of subtype-specific therapeutic strategies in translational cancer research.

## Materials and methods

The protocol described in this peer-reviewed article is published on protocols.io (DOI: dx.doi.org/10.17504/protocols.io.q26g79op8vwz/v1) and is included for printing purposes as S1 File. The S2 File describes the protocol for the LDH assay. The S3 File contains the table of contents. The S4 File presents a comparison of our PI-based flow-cytometry protocol with alternative methods for assessing cell death in organoids.

## Expected results

### Characterization of patient material, GBO treatment and single cell dissociation

**Fig 1:**
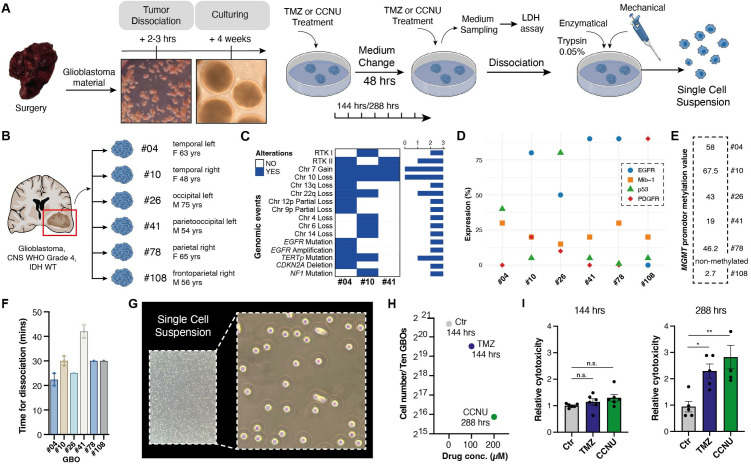
Clinical characteristics of patient material, treatment and dissociation of GBOs into a single cell suspension. **(A)** Visual representation delineating the procedural steps encompassing GBO generation, chemotherapeutic treatment and the dissociation process into a single cell suspension. **(B)** Clinical characteristics from six patients (#04, #10, #26, #41, #78 and #108) whose tumor material was used for generating GBOs (GBO#04, GBO#10, GBO#26, GBO#41, GBO#78, GBO#108). **(C)** Oncoprint plot showing the spectrum of genetic alterations detected in patient-derived tumor material using the 850k methylation array (Illumina) and TruSight Oncology 500 panel (Illumina). **(D)** Quantitative results for EGFR, PDGFR, p53 and MIB-1 expression in parental tumor tissue obtained from immunhistochemistry. **(E)** *MGMT* promoter methylation levels determined by pyrosequencing. **(F)** Barplot illustrating the time required to dissociate GBOs from six different patients into a single cell suspension. **(G)** Brightfield image of an obtained single cell suspension. **(H)** Quantification of total cell count from ten GBOs of GBO#04 under different conditions (Ctr, TMZ and CCNU) and treatment durations (144 hrs and 288 hrs). **(I)** Barplot depicting the relative optical density (OD) values from a cytotoxicity assay, employed to quantify the release of lactate dehydrogenase as an indicator of cytotoxiticity. Higher OD values are reflecting increased cytotoxicity and cell damage. Statistical signiﬁcance was assessed using two-way ANOVA. * and ** denote p < 0.05 and p < 0.01, respectively. Abbreviations: CCNU, lomustine; chr, chromosome; conc, concentration; Ctr, control; EGFR, epidermal growth factor receptor; F, female; GBO, glioblastoma organoid; hrs, hours; IDH, isocitrat dehydrogenase; M, male; *MGMT*, O6-methylguanine-DNA methyltransferase; Mib-1, marker of proliferation Ki-67; mins, minutes; OD, optical density; PDGFR, platelet-derived growth factor receptor; RTK, receptor tyrosine kinase; TMZ, temozolomide; WT, wildtype; yrs, years.

In our study, we adopted the organoid generation protocol described by Jacob et al. [12] as the basis for our translational research (Fig 1A). All patients involved were diagnosed with glioblastoma, CNS WHO Grade 4, IDH Wildtype (Fig 1B) [37]. Analysis of the original tumor tissue revealed a range of molecular alterations, with chromosomal aberrations and mutations (Fig 1C) as well as marked interpatient variability in the expression of EGFR, PDGFR, p53, and MIB-1 (Fig 1D) in immunohistochemical stainings. While most cells were tumor cells, fresh tumor organoids also contained other cell types such as macrophages, endothelial cells, and microglia (S1 Fig). For this study, GBOs from five patients with a hypermethylated *MGMT* promoter, indicative of sensitivity to alkylating chemotherapy, were selected, along with one GBO population from a patient with a glioblastoma with an unmethylated *MGMT* promoter (Fig 1E) (For details on the method used to determine *MGMT* promoter methylation levels, see Schmidt et al. [38]). GBOs were cultivated in suspension on an orbital shaker and underwent regular splitting every one to two weeks to maintain their growth and viability. Treatment was performed with the alkylating chemotherapeutic agents TMZ and CCNU [35,39], mimicking clinical standard of care systemic therapy. To replicate physiological drug concentrations, we exposed GBOs to 100µM of TMZ [40] or CCNU. Establishing a single-cell suspension from GBOs was a pivotal step for flow cytometric analysis of cell death. Multiple methods were explored, and a combination of mechanical and enzymatic dissociation steps proved effective, with trypsin emerging as the most efficient agent for enzymatic dissociation. Notably, the time required for complete dissociation varied based on the specific GBO characteristics like size and density and ranged up to almost 45 mins (Fig 1F, 1G).

### Quantification of treatment effects by cell number and release of lactate dehydrogenase

As an initial indicator of treatment success, we assessed the number of cells in ten GBOs after 144 and 288 hours of treatment with the chemotherapeutic agents TMZ and CCNU (Fig 1H). A decrease in cell numbers post-treatment and fewer cells after prolonged exposure (144 versus 288 hours) indicated induced treatment effects. Furthermore, we measured treatment-induced effects using a cytotoxicity assay which detects the LDH release of damaged cells (Fig 1I, S2B Fig).

Drug exposures were conducted over 144 hours initially, considering our previous data from monolayer cell cultures [23,24,36]. Our observations revealed that LDH release after 144 hours of drug exposure lacked statistical significance when measured from supernatants collected following 48-hourly medium exchange. Notably, GBOs have a substantially higher density compared to monolayer cultures, impacting the distribution of the drug-containing medium. Thus, we extended the exposure duration to 288 hours to adjust for these variances in medium perfusion. After the extended exposure period of 288 hours, a significant increase in LDH release was evident for treatment with TMZ and CCNU (relative optical density (OD) values for TMZ: 2.33, CCNU: 2.83 compared to 1 in the control (Ctr), p = 0.01 and p = 0.008 for GBO#10; TMZ: 2.8 compared to 1 in the control for GBO#78, p = 0.03). However, LDH measurements are less precise and can be heavily influenced by the number of cells per GBO before treatment was administered as toxicity of one treatment condition can be overestimated when GBOs with more cells were treated compared to another condition. Therefore, a more specific method is needed.

### Flow cytometric analysis of chemotherapy-induced cell death in GBOs using propidium iodide staining

**Fig 2:**
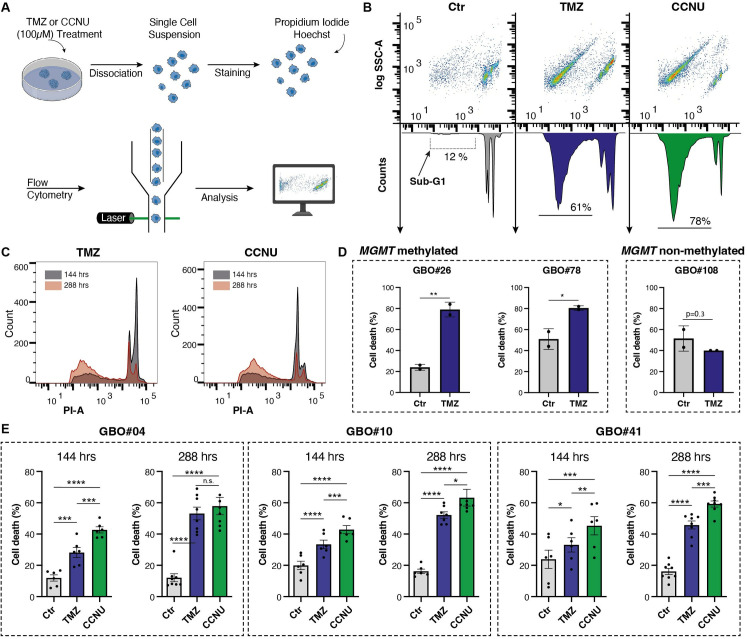
Assessing cell death by measuring the DNA fragmentation of propidium iodide-stained nuclei of GBOs subjected to chemotherapeutic treatment. **(A)** Visual representation outlining the workflow for conducting flow cytometric analysis to assess cell death rates after chemotherapeutic treatment using propidium iodide (PI) staining. **(B)** Representative density plots and histograms derived from flow cytometric analysis of GBO#10, 288 hours after treatment start. The sub-G1 peak is accentuated within the histograms and cell death rates are depicted below. **(C)** Overlays of histograms contrasting the effects of TMZ and CCNU treatment durations (144 hrs and 288 hrs) for GBO#10. **(D)** Barplots illustrating the response to TMZ treatment stratified by *MGMT* promoter methylation status (methylated: GBO#26, GBO#78; unmethylated: GBO#108). **(E)** Barplots depicting the cell death rates after treatment with TMZ (blue) and CCNU (green) for a treatment duration of 144 hrs (left bars) and 288 hrs (right bars) for GBO#04, #10 and #41, results for staining with PI (1 mg/ml). Statistical signiﬁcance was assessed using two-way ANOVA. *, **, *** and **** denote p < 0.05, p < 0.01, p < 0.001 and p < 0.0001, respectively. Abbreviations: CCNU, lomustine; Ctr, control; GBO, glioblastoma organoid; hrs, hours; *MGMT*, O6-methylguanine-DNA methyltransferase; PI, propidium iodide; PI-A, PI-Area; SSC-A, side scatter area; TMZ, temozolomide.

A robust and accurate method for assessing cell death is flow cytometry [32]. For each treatment condition, ten GBOs of approximately equal size were selected, and the drug-containing medium was replaced every two days. Here, we could prove that flow cytometry is a highly effective method for measuring cell death within the GBO model. We utilized PI as a marker to assess cell death following treatment with chemotherapeutic agents by detecting the fragmentation of DNA within individual cells (Fig 2A). Triton-X was employed for cellular permeabilization, enabling the exit of low-molecular-weight DNA from the nucleus. Hypodiploid cells with diminished cellular DNA content are retained in the case of cell death. The removal of RNA occurred during the hypotonic shock treatment. In our study, we noted that, beyond observations in various cell lines, PI staining facilitated the measurement of hypodiploid cells within the sub-G1 peak in our GBO model (Fig 2B) [41]. During data analysis, debris, nuclear fragments and clumps of chromosomes were gated-off since they exhibited minimal DNA fluorescence and reduced diameter. As expected, treatment with different chemotherapeutic agents and measurement at different time points could show significantly different cell death rates (Fig 2C–2E). Exposure to chemotherapeutic agents led to increased cell death, as indicated by DNA fragmentation in PI-stained nuclei, in *MGMT*-methylated GBOs but not in *MGMT*-unmethylated GBOs (Fig 2D). The lack of TMZ sensitivity in *MGMT*-unmethylated GBO#108 was further supported by the absence of a significant increase in LDH release after 288 hours of treatment (S2B Fig). At 144 hours after treatment initiation, increased cell death was observed in the *MGMT*-methylated GBOs (e.g., 33% for TMZ and 43% for CCNU, compared to 20% in the untreated control group in GBO#10 (p < 0.0001)). However, an increase in treatment effects became apparent when exposure duration was extended to 288 hours (e.g., 33% to 52% for TMZ, 43% to 63% for CCNU in GBO#10 without significant differences in spontaneous cell death rates in the Ctr, p = 0.0012 for 144 vs. 288 hrs) (Fig 2C, 2E). Interestingly, GBO#04 and GBO#10 populations, which exhibited the highest levels of *MGMT* promoter methylation, demonstrated the most pronounced response to TMZ treatment among all tested GBOs. CCNU treatment consistently resulted in higher cell death rates across all tested patients and both timepoints at the given concentration (Fig 2E). To compare treatment effects across GBOs, we calculated relative cell death by normalizing TMZ-induced cell death to the respective untreated control (TMZ/Ctr). In line with our expectations, we observed interindividual differences in the response of GBOs to treatment at 288 hours after treatment initiation (GBO#04: 4.4, GBO#10: 3.3, GBO#26: 2.2, GBO#41: 3.0, GBO#78: 1.5, GBO#108: 0.8). This highlighted the existence of interindividual heterogeneity, and our method proved capable of capturing these distinct treatment effects. Furthermore, low variability across technical and biological replicates indicates high reproducibility (Fig 2D, 2E).

Majc et al. [14] examined treatment-induced effects in GBOs using an Annexin V/PI protocol and reported only very small TMZ effects, limited to a subset of organoids. In contrast, we observed substantially larger effects, likely because their exposure was restricted to one week at 50 µM TMZ. Step-by-step details required for replication are not provided, as this was not the focus of their manuscript, limiting straightforward reproduction by external laboratories.

### Hoechst 33258 staining

**Fig 3:**
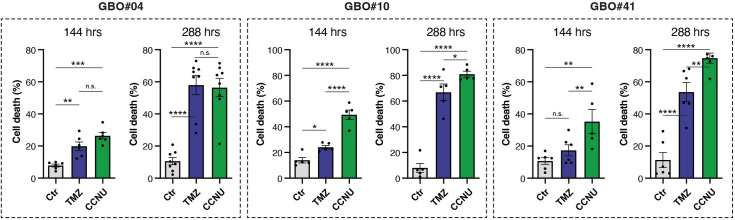
Quantification of cell death for TMZ and CCNU treatment at two timepoints. Barplots depicting the cell death rates after treatment with TMZ (blue) and CCNU (green) for a treatment duration of 144 hrs (left bars) and 288 hrs (right bars) for GBO#04, #10 and #41, results for staining with Hoechst 33258. Statistical signiﬁcance was assessed using two-way ANOVA. *, **, *** and **** denote p < 0.05, p < 0.01, p < 0.001 and p < 0.0001, respectively. Abbreviations: CCNU, lomustine; Ctr, control; GBO, glioblastoma organoid; hrs, hours; PI, propidium iodide; TMZ, temozolomide.

To validate the reliability of the PI staining, we conducted an additional staining procedure utilizing Hoechst and subsequently compared the results with those derived from PI staining on the same samples (Fig 3, S2A Fig). Numerous Hoechst dyes exist with distinct chemical structures, leading to variations in their properties such as cell permeability and fluorescence emission. These dyes are widely employed for DNA staining in both living and fixed cells, each being chosen for specific applications based on its unique characteristics [30,42]. Hoechst 33258 is known for its affinity to AT-rich regions in double-strand DNA (dsDNA) and is commonly used for assessing cellular viability in various cell types [43]. The damage induced by chemotherapy often compromises cellular membrane integrity, leading to increased dye intake [44]. This process permits Hoechst dye to enter dying cells and mark their DNA. Upon binding to dsDNA, it enhances emission levels, which can be detected and quantified as a marker for dead cells. Our results, obtained through Hoechst staining, cross-verified the observed trends in cell death for CCNU and TMZ, as well as the two measuring time points derived from PI staining (Fig 3). Moreover, all *MGMT*-methylated GBOs showed a clear response to chemotherapy, whereas no such effect was seen for TMZ in the *MGMT*-unmethylated GBO#108. Consistent with the PI data, GBO#04 and GBO#10 showed the highest TMZ-induced cell death rates, thereby further supporting the robustness and consistency of our results (S2A Fig).

### Measurement of organoid diameter to confirm baseline equivalence and quantify treatment-induced changes

**Fig 4:**
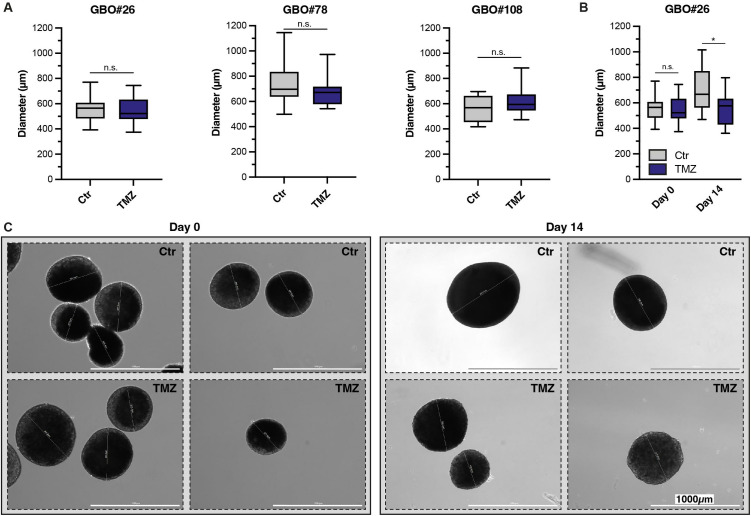
Assessment of GBO diameter using live-cell imaging before and after treatment. **(A)** Boxplots showing the diameters of GBO#26, GBO#78 and GBO#108 before treatment. **(B)** Boxplots depicting the diameters of GBO#26 before (Day 0) and after 14 days of treatment in control and TMZ-treated groups. **(C)** Representative phase-contrast images acquired using the Lionheart FX live-cell imaging system at baseline (Day 0) and after treatment (Day 14) for both control and TMZ-treated GBOs. Statistical signiﬁcance was assessed using Mann-Whitney test. * and ** denote p < 0.05 and p < 0.01, respectively. Abbreviations: Ctr, control; GBO, glioblastoma organoid; TMZ, temozolomide.

Since direct cell counting prior to treatment would require dissociation of the organoids, which we aimed to avoid, we instead measured the diameter of the organoids to ensure equal size distribution between treated and untreated groups (Fig 4A–4C). At day 0, the average diameters did not differ significantly between control and TMZ groups (GBO#26: p = 0.88, GBO#78: p = 0.22, GBO#108: p = 0.27), indicating comparable baseline conditions (Fig 4A). By day 14, untreated GBO populations increased in diameter, as expected. For GBO#26, the mean diameter (± SD) was 550 ± 85 µm at day 0 and increased to 698 ± 156 µm at day 14 (Fig 4B), corresponding to an approximate 27% increase (≈1.27-fold). Under TMZ treatment, a slight reduction in mean diameter was observed over 14 days (day 0: 553 ± 99 µm; day 14: 542 ± 120 µm; p = 0.01), corresponding to an approximate 2% decrease (≈0.98-fold). These treatment-induced differences in cell number within the GBOs further supported the results obtained from flow cytometric analysis.

## Supporting information

S1 File Protocol for flow cytometric analysis for protocols.io. (PDF)

S2 File Protocol for LDH assay. (PDF)

S3 File Table of materials. (PDF)

S4 File Comparative overview of assays for quantifying treatment-induced cell death in organoids. (PDF)

S1 Fig Immunohistochemistry of GBO material. Immunohistochemical staining was performed on GBO#26, GBO#78, and GBO#108 using hematoxylin and eosin (H&E), CD31 (marker for endothelial cells and macrophages), CD45 (marker for leukocytes), and KP1 (marker for macrophages and activated microglia). Abbreviations: GBO, glioblastoma organoids.
(TIF)

S2 Fig Cell death rates from Hoechst staining and relative cytotoxicity from LDH assay. (A) Barplot showing cell death rates from flow cytometry experiments based on Hoechst staining in GBO#26 and GBO#108. (B) Relative cytotoxicity values from the LDH assay for GBO#26 following TMZ treatment. * and ** denote p < 0.05 and p < 0.01, respectively. Abbreviations: Ctr, control; GBO, glioblastoma organoid; TMZ, temozolomide.
(TIF)
