# Supplementary material for: Flow cytometry protocol for cell death analysis in glioblastoma organoids: A technical note
Source: PLoS One. 2025 Sep 23;20(9):e0327660. doi: 10.1371/journal.pone.0327660 (PMC12456761; doi:10.1371/journal.pone.0327660)
Supplement: S1 File — (PDF) [file pone.0327660.s001.pdf]

Sep 15, 2025

Flow Cytometry Protocol for Cell Death Analysis in Glioblastoma Organoids: A Technical Note

DOI
dx.doi.org/10.17504/protocols.io.q26g79op8vwz/v1

Anna-Laura Potthoff[1,2,3], Meng-Chun Hsieh[1,2], Ahmad Melhem[1,2], Susanna S. Ng[4], Barbara E. F. Pregler[1,2], Annika Vieregge[1,2], Markus Raspe[1,2], Lea L. Friker[2,3,4], Thomas Zeyen[2,5], Julian P. Layer[2,4,6], Andreas Dolf[7], Marieta I. Toma[8], Andreas Waha[3], Torsten Pietsch[3], Mike-Andrew Westhoff[9], Hartmut Vatter[1], Michael Hölzel[4], Ulrich Herrlinger[2,5], Matthias Schneider[1,2]

[1]Department of Neurosurgery, University Hospital Bonn, Bonn, Germany;
[2]Brain Tumor Translational Research Group, University Hospital Bonn, Bonn, Germany;
[3]Institute of Neuropathology, University Hospital Bonn, Bonn, Germany;
[4]Institute of Experimental Oncology, University Hospital Bonn, Bonn, Germany;
[5]Department of Neurooncology, Center for Neurology, University Hospital Bonn, Bonn, Germany;
[6]Department of Radiation Oncology, University Hospital Bonn, Bonn, Germany;
[7]Flow Cytometry Core Facility, Medical Faculty, University of Bonn, Bonn, Germany;
[8]Department of Pathology, University Hospital Bonn, Bonn, Germany;
[9]Department of Pediatrics and Adolescent Medicine, University Medical Center Ulm, Ulm, Germany

Anna-Laura Potthoff
University Hospital Bonn, Department of Neurosurgery

OPEN ACCESS

DOI: dx.doi.org/10.17504/protocols.io.q26g79op8vwz/v1

Protocol Citation: Anna-Laura Potthoff, Meng-Chun Hsieh, Ahmad Melhem, Susanna S. Ng, Barbara E. F. Pregler, Annika Vieregge, Markus Raspe, Lea L. Friker, Thomas Zeyen, Julian P. Layer, Andreas Dolf, Marieta I. Toma, Andreas Waha, Torsten Pietsch, Mike-Andrew Westhoff, Hartmut Vatter, Michael Hölzel, Ulrich Herrlinger, Matthias Schneider 2025. Flow Cytometry Protocol for Cell Death Analysis in Glioblastoma Organoids: A Technical Note. **protocols.io** **https://dx.doi.org/10.17504/protocols.io.q26g79op8vwz/v1**

License: This is an open access protocol distributed under the terms of the **Creative Commons Attribution License**, which permits unrestricted use, distribution, and reproduction in any medium, provided the original author and source are credited

Protocol status: Working
We use this protocol and it's working

Created: June 10, 2025

Last Modified: September 15, 2025

Protocol Integer ID: 220179

Keywords: Flow cytometry protocol, Cell death analysis, Glioblastoma organoids, flow cytometry protocol for cell death analysis, cell death in glioblastoma organoid, flow cytometry protocol, using flow cytometry, flow cytometry, glioblastoma organoid, throughput applications in translational cancer research, cell death analysis, flow cytometric analysis, cytometric analysis, translational cancer research, cell death rate, extent of cell death, cell death, dna fragmentation, cell suspension

Abstract

Here, we present a protocol to quantify cell death in glioblastoma organoids (GBOs) using flow cytometry. Single-cell suspensions are generated through combined enzymatic and mechanical dissociation, followed by permeabilization and nuclear staining with propidium iodide or Hoechst 33258 to detect DNA fragmentation. Following treatment with temozolomide (TMZ) and lomustine (CCNU) for up to 288 hours, flow cytometric analysis revealed cell death rates of up to 63%, with CCNU demonstrating greater efficacy than TMZ. The extent of cell death varied between GBO populations but was consistent across replicates derived from the same GBO population. The protocol is scalable, cost-effective, and suitable for high-throughput applications in translational cancer research.

Guidelines

Protocol for flow cytometric analysis

Here, we provide a protocol for measuring cell death in GBOs after treatment with two different chemotherapeutic drugs. For us, measurement time points 144 hours and 288 hours after treatment initiation are sufficient. However, depending on desired drugs, earlier measurement time points might be more suitable. The protocol can be adapted for organoids from other models or tumor entities. Therefore, parameters such as the number of organoids needed, incubation times, and other steps need to be adjusted. We recommend processing no more than 12 samples at a time to ensure consistency and manageability. Within the combined enzymatic and mechanical dissociation step, it is important to resuspend the suspension and observe it under the microscope every few minutes to monitor the progress of the dissociation process.

Materials

Table of materials

A	B	C	D
**Reagent name**	**Manufacture**	**Category number**	**Note**
GBO cultured medium			
DMEM/F12	Thermo Fisher Scientific	11320033	
Neurobasal medium	Thermo Fisher Scientific	21103049	
MEM-NEAAs solution (100x)	Thermo Fisher Scientific	11140050	
GlutaMAX supplement (100x)	Thermo Fisher Scientific	35050061	
N2 supplement (100x)	Thermo Fisher Scientific	17502048	
B27 supplement (50x), minus vitamin A	Thermo Fisher Scientific	12587010	
Penicillin-streptomycin (100x)	Thermo Fisher Scientific	15070063	
Human insulin solution	Sigma-Aldrich	I9278	
2-Mercaptoethanol	Thermo Fisher Scientific	21985023	1:1000 dilution in GBO medium

- DMEM/F12 **Thermo Fisher Scientific Catalog #**11320033
- Neurobasal™ Medium **Gibco - Thermo Fisher Scientific Catalog #**21103049
- MEM Non-Essential Amino Acids **Gibco - Thermo Fisher Scientific Catalog #**11140050
- GlutaMAX™ Supplement **Gibco - Thermo Fisher Scientific Catalog #**35050061
- N-2 Supplement (100X) **Thermo Fisher Catalog #**17502048
- B-27™ Supplement (50X) minus vitamin A **Thermo Fisher Scientific Catalog #**12587010
- Penicillin-Streptomycin (5,000 U/mL) **Thermo Fisher Catalog #**15070063
- Insulin solution human **Merck MilliporeSigma (Sigma-Aldrich) Catalog #**I9278
- 2-Mercaptoethanol **Gibco - Thermo Fisher Scientific Catalog #**21985023

A	B	C	D
PI buffer			
Propidium iodide (PI) stock (1mg/1ml)	SIGMA	P4170	1mg/ml stock in Ampuwa water dilution

A	B	C	D
Triton X-100	SIGMA	T8787	0.10%
Trisodium citrate	Merck KGaA	1.37042.1000	0.10%

- Propidium Iodide (PI) **Merck MilliporeSigma (Sigma-Aldrich) Catalog #**P4170
- Triton X-100 **Merck MilliporeSigma (Sigma-Aldrich) Catalog #**T8787

A	B	C	D
**Reagent name**	**Manufacture**	**Category number**	**Note**
Trypsin-EDTA (0.5%)	gibco	15400054	no phenol red
CytoTox 96® Non-Radioactive Cytotoxicity Assay	Promega	G1780	
Hoechst 33258	SIGMA	B1155_25MG	10 mg/ml stock in Ampuwa water dilution

- Trypsin-EDTA (0.5%) no phenol red **Thermo Fisher Scientific Catalog #**15400054
- CytoTox 96(R) Non-Radio. Cytotoxicity Assay, 10X96 well plt **Promega Catalog #**G1780

A	B	C	D
**Drug name**	**Manufacture**	**Category number**	**Note**
Temozolomide (TMZ)	TOCRIS	2706	
Lomustine (CCNU)	Merck	L5918	

A	B	C	D
**Equipment name**	**Manufacture**	**Category number**	**Note**
6 well-plate (suspension)	Sarstedt	83.3920.500	
1000ul filtered tip	Sarstedt	70.3060.255	
Eppendorf® Combitips Advanced® 5ml	Eppendorf	30089456	
15ml falcon	Thermo Fisher Scientific	339650	
50ml falcon	Thermo Fisher Scientific	339652	
Cell Strainer 70µm	Corning	7201431	
Flow Cytometry Tube 5 ml	Sarstedt	55.1579	75 × 12 mm, PS
Orbital shaker	Thermo Fisher Scientific		
Magnetic stirrer			

A	B	C	D
FACS Canto II	Becton Dickinson, Heidelberg, Germany		
Centrifuge 5810 R	Eppendorf		
µQuant microplate spectrophotometer	Biotek Instruments, USA		

A	B	C	D
**Software name**	**Manufacture**	**Category number**	**Note**
FlowJo	FlowJo LLC, Ashland, OR, USA		V10.4
GraphPad PRISM	GraphPad Software, Boston, M, USA		V9.5.1
Adobe Illustrator 2023	Adobe Inc., Dublin Republic of Ireland		V27.7

Treatment (Day 0)

1 **Preparation of GBO Culture Medium**

1.1 Prepare the GBO culture medium as previously described by Jacob et al. [1]. The medium consists of a 1:1 mixture of DMEM:F12 medium (235 mL) and Neurobasal medium (235 mL), supplemented with the following components:

- 5 mL of MEM-NEAAs solution (100×),
- 5 mL of GlutaMAX (100×),
- 5 mL of penicillin-streptomycin (100×),
- 5 mL of N2 supplement (100×),
- 10 mL of B27 minus vitamin A (50×),
- 125 µL of human recombinant insulin.

1.2 Sterilize the medium by filtration through a 0.2-µm PES membrane. Immediately before use, add 2-mercaptoethanol at a 1:1000 dilution. Refer to the material table for additional details.

Note

- The complete GBO medium (without 2-mercaptoethanol) can be stored at 4 °C for up to 4 weeks.
- 2-mercaptoethanol must be added to the GBO medium just before use.

2 **GBO Selection and Treatment Setup**

2.1 **GBO Selection**:

- Select 8-10 GBOs per treatment condition based on size (optimal size range: 500–700 µm).
- Ensure all selected GBOs are of approximately similar size to avoid inconsistencies in drug penetration and inaccurate results.

- Measure the diameter of all GBOs and calculate the mean diameter per treatment condition to ensure there is no significant difference between groups. If the organoids are not perfectly round, record the longest diameter.

2.2 **Plate Setup:**

- Transfer the selected 8-10 GBOs to individual wells of a non-coated 6-well plate, with each treatment condition assigned to a separate well.
- Perform at least technical duplicates (i.e., two wells per condition) to ensure reproducibility.

Note

- For better control and safety, prepare extra wells for flow cytometry acquisition if performing the procedure for the first time or using new GBOs populations (new patient material).
- Always include an untreated control group to assess the level of spontaneous cell death (usually higher in GBOs than in 2D cell culture populations).

2.3 **Medium Addition:**

- Add 4 mL of GBO culture medium to the control wells.
- Add 4 mL of GBO medium containing the desired drug concentration to the treatment wells (TMZ/CCNU 100µM concentration: 1:1000 from a [M] 100 millimolar (mM) stock (4 µL for 4 mL)).

2.4 **Incubation:**

Place the 6-well plate on an orbital shaker set to 120 rpm inside a humidified incubator (37 °C , 5% CO_2, 90% relative humidity).

Medium Change (Every 48 hours)

2m

3 The medium must be changed every 48 hours due to the high cell density within the GBOs and the rapid proliferation rate of individual cells.

3.1 **Removal of Old Medium:**

2m

- Remove the 6-well plate from the incubator.

- Allow the organoids to settle by tilting the plate at a slight angle for 1– 00:02:00 .
- Gently aspirate the old medium from each well using a 1000 µl filtered tip and transfer it into a 15 ml centrifuge tube.
- Store the centrifuge tube containing the collected medium at -20 °C (collected medium will be used later for flow cytometry and optionally for the LDH assay).

Note

- Not including the old medium in flow cytometry measurement may lead to underestimation of cell death, as dead cells may detach and be in suspension.
- GBOs are sticky and may adhere to the pipette tip during medium change. If any GBO adhered to the tip, reload it back into the well and re-do the procedure.

3.2 **Addition of Fresh Medium:**

- Add 4 mL of fresh drug-containing medium to each well, as described in Step 2.3.

Note

- Repeat the medium change every 48 hours until the designated measurement time point is reached.
- Pool the collected medium from each condition into the corresponding centrifuge tube and store it at -20 °C .

GBO Dissociation for Subsequent PI Staining and Cell Death Measurement at the Designated Time Point

25m

4 **Preparation of propidium iodide (PI) Buffer:**

- Combine 5 mL of PI stock (1 mg/ml) with 95 mL of 0.1% sodium citrate to achieve a final concentration of 50 mg/l.
- Add 100 µL of Triton X-100 (0.1%) and mix thoroughly on a magnetic stirrer until complete dissolution is achieved.

Note

- You may prepare the buffer one day in advance.
- Cover the PI buffer container with aluminum foil to protect it from light.
- Store at 4 °C in darkness. The buffer can be stored for up to 6 months.
- Always wear gloves when handling PI buffer, as it is mutagenic.

Additional Preparations:

- Pre-warm trypsin in a water bath to 37 °C .
- Prepare an ice bucket.

4.1 **Thawing Collected Medium:**

On the day of measurement, thaw the centrifuge tubes containing the collected medium by placing them at Room temperature .

Note

Start thawing 1–2 hrs in advance, and place the centrifuge tubes On ice once thawed.

4.2 **Collection of Final Medium:**

Collect the last medium from the wells and transfer it to the corresponding thawed centrifuge tubes.

Note

Remove as much medium as possible from each well. If necessary, use a smaller tip to remove the remaining medium without aspirating GBOs.

4.3 **Washing GBOs:**

Wash the GBOs with 2 mL of DPBS to remove any residual medium. Gently tilt the plate back and forth and side to side a few times. Discard the DPBS and repeat the wash

step again.

4.4 **Enzymatic Dissociation:**

10m

- Add 2 mL of warm trypsin (0.05%) to each well for enzymatic dissociation. Tilt the plate or gently swirl to ensure that the GBOs are completely immersed.
- Place the plate on the orbital shaker in the incubator at 37 °C for 00:10:00 .

4.5 **Centrifugation of Collected Medium:**

10m

- While waiting, centrifuge all thawed centrifuge tubes at 4000 rpm, 4°C, 00:10:00 .
- Discard the supernatant until 3 ml remains in each centrifuge tube. Keep the tubes On ice .

4.6 **Mechanical Dissociation:**

- After 10 minutes, speed up the dissociation process by gently pipetting up and down 5–10 times using a wide-bored 1000 µl pipette tip.
- Repeat mechanical dissociation every 5-10 minutes, returning the plate to the incubator between steps.

Note

- Enzymatic dissociation alone is insufficient; combine it with gentle mechanical dissociation to effectively and rapidly reach single-cell suspension.
- Avoid vigorous pipetting to prevent cell damage or death.

4.7 **Monitoring Dissociation:**

Monitor dissociation progress under a microscope after each resuspension step until no cell clumps are visible.

Note

- Dissociation time varies with GBO size and density, and between different GBO populations (different patient material).
- Avoid over-trypsinization; for tested GBOs, the mean dissociation time was ~ 00:30:00 (range: 20–45 minutes).

4.8 **Filtration:**

- **Filter** the cell suspension through a 70 µm strainer to avoid small cell aggregates.

4.9 **Neutralization of Trypsin:**

- **Transfer** the filtered single-cell suspension to the corresponding centrifuge tube containing 3 ml of supernatant (from Step 4.5) to slow the trypsin reaction.

4.10 **Addition of HBSS (optional):**

- Add 2 mL of HBSS to the centrifuge tubes to maintain osmolality and pH.

4.11 **Centrifugation:** 5m

- Centrifuge at 1300 rpm, 4°C, 00:05:00 .

4.12 **Discarding Supernatant:**

- Gently discard the supernatant by pouring. Some residual medium is acceptable.

4.13 **Resuspension:**

- Resuspend the cell pellet in the remaining volume (typically 300–350 µl) in the 15 ml centrifuge tube.

Staining of Single Cell Suspension with PI-Buffer 1h

5 **Transfer Single Cell Suspension:**

- Transfer the entire single-cell suspension into a FACS tube (round-bottom FACS tube for BD FACS Canto™ II, BD Biosciences).

6 **Add HBSS:**

- Add 100 µL HBSS to prevent aggregation and maintain osmolality.

7 **Divide Cell Suspension:**

- Divide the single-cell suspension equally into two FACS tubes (approx. 150 µL per tube), one for PI staining and the other one for Hoechst staining **(see Step 11).**

8 **Add PI-Buffer:**

- Add 100 µL of the PI-buffer to each tube.

9 **Incubation:**

30m

- Incubate the tubes On ice for 20- 00:30:00 .

Note

- Perform flow cytometric measurement within 4 hours after adding PI-buffer. For best results, start measurement immediately after incubation and complete within 1 hour to prevent cell aggregation.
- Keep all samples On ice until measurement.

10 **Data acquisition:**

10.1 Set up the flow cytometry device (e.g., BD FACSCanto™ II, Becton Dickinson) for analysis.

10.2 Suggested settings (may vary between organoid model/GBO population and device): FCS threshold: 800; Voltages: SSC: 243, FCS: 132, PI: 291.

Note

- Set an accurate threshold to exclude debris while ensuring the sub-G1 peak (containing dead cell information) is not cut off.
- Check for doublets while acquisition (e.g., plot FSC-A vs. FSC-H).

Staining of Single Cell Suspension with Hoechst-Buffer

30m

11 **Preparation of Hoechst Buffer**

- Dilute Hoechst 33258 in HBSS to achieve a 1:5000 dilution ratio.

11.1 **Add Hoechst Buffer:**

- Add the same volume of Hoechst buffer to the single-cell suspension to achieve a final dilution of 1:10000 (150 µL in this protocol).

11.2 **Incubation:**

30m

- Incubate On ice for 15– 00:30:00 .

11.3 **Data acquisition:**

- Settings vary between organoid model/GBO population and device (example settings for our GBO populations and BD FACS Canto II, BD Biosciences: FCS threshold: 800; Voltages: SSC: 243, FCS: 132, 450/50 nm violet: 269.

Note

- Use the same settings for threshold as well as SSC and FSC voltages as applied during the PI measurement.

Protocol references

1. Jacob F, Ming GL, Song H. Generation and biobanking of patient-derived glioblastoma organoids and their application in CAR T cell testing. Nature protocols. 2020;15(12):4000-33. Epub 20201109. doi: 10.1038/s41596-020-0402-9. PubMed PMID: 33169003.
