# Supplementary material for: Flow cytometry protocol for cell death analysis in glioblastoma organoids: A technical note
Source: PLoS One. 2025 Sep 23;20(9):e0327660. doi: 10.1371/journal.pone.0327660 (PMC12456761; doi:10.1371/journal.pone.0327660)
Supplement: S2 File — (PDF) [file pone.0327660.s002.pdf]

Supporting information: S2 file

Flow cytometry protocol for cell death analysis in glioblastoma organoids: a technical note

Anna-Laura Potthoff, Meng-Chun Hsieh, Ahmad Melhem, Susanna S. Ng, Barbara E. F. Pregler, Annika Vieregge, Markus Raspe, Lea L. Friker, Thomas Zeyen, Julian P. Layer, Andreas Dolf, Marieta I. Toma, Andreas Waha, Torsten Pietsch, Mike-Andrew Westhoff, Hartmut Vatter, Michael Hölzel, Ulrich Herrlinger, Matthias Schneider

Protocol for LDH assay

CytoTox 96® Reagent Preparation:

- Combine the substrate mix and assay buffer according to the manufacturer's instructions.
- Store the prepared reagent at -20 °C until use.

Procedure:

1. **Sample Dispensing:**
   - Dispense 50 µl of the supernatant sample into each well of a 96-well plate. Ensure triplicates for each sample.

2. **Reagent Addition and Incubation:**
   - Add 50 µl of CytoTox 96® Reagent to each well.
   - Incubate the plate for 30 minutes in a dark environment at room temperature.

3. **Stop Reaction:**
   - Add 50 µl of stop solution to each well to terminate the reaction.

4. **Absorbance Measurement:**
   - Measure the absorbance at 490 nm using a microplate spectrophotometer (e.g., µQuant, BioTek Instruments, USA).
