# Supplementary material for: Flow cytometry protocol for cell death analysis in glioblastoma organoids: A technical note
Source: PLoS One. 2025 Sep 23;20(9):e0327660. doi: 10.1371/journal.pone.0327660 (PMC12456761; doi:10.1371/journal.pone.0327660)
Supplement: S3 File — (PDF) [file pone.0327660.s003.pdf]

Supporting information: S3 file

Flow cytometry protocol for cell death analysis in glioblastoma organoids: a technical note

Anna-Laura Potthoff, Meng-Chun Hsieh, Ahmad Melhem, Susanna S. Ng, Barbara E. F. Pregler, Annika Vieregge, Markus Raspe, Lea L. Friker, Thomas Zeyen, Julian P. Layer, Andreas Dolf, Marieta I. Toma, Andreas Waha, Torsten Pietsch, Mike-Andrew Westhoff, Hartmut Vatter, Michael Hölzel, Ulrich Herrlinger, Matthias Schneider

Table of materials

Reagent name	Manufacture	Category number	Note
GBO cultured medium			
DMEM/F12	Thermo Fisher Scientific	11320033	
Neurobasal medium	Thermo Fisher Scientific	21103049	
MEM-NEAAs solution (100x)	Thermo Fisher Scientific	11140050	
GlutaMAX supplement (100x)	Thermo Fisher Scientific	35050061	
N2 supplement (100x)	Thermo Fisher Scientific	17502048	
B27 supplement (50x), minus vitamin A	Thermo Fisher Scientific	12587010	
Penicillin-streptomycin (100x)	Thermo Fisher Scientific	15070063	
Human insulin solution	Sigma-Aldrich	I9278	
2-Mercaptoethanol	Thermo Fisher Scientific	21985023	1:1000 dilution in GBO medium
PI buffer			
Propidium iodide (PI) stock (1mg/1ml)	SIGMA	P4170	1mg/ml stock in Ampuwa water dilution

Triton X-100	SIGMA	T8787	0.1%
Trisodium citrate	Merck KGaA	1.37042.1000	0.1%

Reagent name	Manufacture	Category number	Note
Trypsin-EDTA (0.5%)	gibco	15400054	no phenol red
CytoTox 96® Non-Radioactive Cytotoxicity Assay	Promega	G1780	
Hoechst 33258	SIGMA	B1155_25MG	10mg/ml stock in Ampuwa water dilution

Drug name	Manufacture	Category number	Note
Temozolomide (TMZ)	TOCRIS	2706	
Lomustine (CCNU)	Merck	L5918	

Equipment name	Manufacture	Category number	Note
6 well-plate (suspension)	Sarstedt	83.3920.500	
1000ul filtered tip	Sarstedt	70.3060.255	
Eppendorf® Combitips Advanced® 5ml	Eppendorf	30089456	
15ml falcon	Thermo Fisher Scientific	339650	
50ml falcon	Thermo Fisher Scientific	339652	
Cell Strainer 70μm	Corning	7201431	

Flow Cytometry Tube 5 ml	Sarstedt	55.1579	75 x 12 mm, PS
Orbital shaker	Thermo Fisher Scientific		
Magnetic stirrer			
FACS Canto II	Becton Dickinson, Heidelberg, Germany		
Centrifuge 5810 R	Eppendorf		
µQuant microplate spectrophotometer	Biotek Instruments, USA		
**Software name**	**Manufacture**	**Category number**	**Note**
FlowJo	FlowJo LLC, Ashland, OR, USA		V10.4
GraphPad PRISM	GraphPad Software, Boston, M, USA		V9.5.1
Adobe Illustrator 2023	Adobe Inc., Dublin Republic of Ireland		V27.7
