# Supplementary material for: Flow cytometry protocol for cell death analysis in glioblastoma organoids: A technical note
Source: PLoS One. 2025 Sep 23;20(9):e0327660. doi: 10.1371/journal.pone.0327660 (PMC12456761; doi:10.1371/journal.pone.0327660)
Supplement: S4 File — (PDF) [file pone.0327660.s004.pdf]

Method	Mechanism	Advantage	Disadvantage	Device needed	Costs*	Time**	Sensitivity	Specificity	Throughput	Information content
**Sulforhodamine B**	Stoichiometric binding of SRB dye to cellular proteins; extracted dye is a proxy for cell mass/cell number	Cost-efficient, relatively fast	Mixed readout of viability, cytotoxicity, and proliferation, no distinction between apoptosis vs necrosis	Multi-well spectrophotometer	€400 per 1,000 tests	~3 h total (≈1.5 h hands-on)	7	3	9	4
**Adenosintriphosphat (ATP) Assay *** **	Luciferase-based luminescence proportional to ATP in metabolically active cells	Cost-efficient, fast	Reports metabolic activity (ATP) rather than death mode, susceptible to compounds that alter ATP or quench luminescence	Luminometer	€550 per 1,000 tests	~1.5 h	7	3	9	4
**PI/Triton-X (flow cytometry)**	Triton X permeabilize cells, PI intercalates nuclear DNA; apoptosis-related DNA fragmentation appears as a hypodiploid sub-G1 peak	Cost-efficient, relatively fast, enables DNA-content–based cell-cycle analysis	Prior training/introduction in flow cytometry is necessary, does not distinguish apoptosis vs necrosis	Centrifuge, flow cytometer	€50 per 1000 tests	~4h (≈1,5 h hands-on)	8	8	6	7
**Annexin V/Propidium iodide (PI) (flow cytometry)**	Annexin V binds externalized phosphatidylserine (early apoptosis); PI enters cells with compromised membranes and intercalates DNA (late apoptosis/necrosis)	Discriminates early vs late apoptosis and necrosis, allows for multiplexing with other dyes	More complex setup and analysis and requires more experience than PI mono-staining; more expensive	Centrifuge, flow cytometer	€400 per 100 samples	~4h (≈1,5 h hands-on)	8	8	6	8
**Live cell imaging (brightfield/phase-contrast)**	Measure cell/organoid diameter/area over time from time-lapse images	Longitudinal observation; cost-neutral if the device is available	No discrimination between proliferation, cell death, or viability	Live-cell imaging microscope (ideally with option to incubate)	€ 0	~30min hands-on	4	3	5	4
**Caspase 3/7 live-cell dye**	Cell-permeant caspase-3/7 substrates that become fluorescent upon enzymatic cleavage (early-apoptosis readout)	Live-cell, kinetic readout, compatible with high-content imaging	Lacks caspase-independent death detection; limited penetration in larger organoids	microplate reader for bulk readout or other microscope	€400 per 100 samples	~30min hands-on	5	6	7	5
**TUNEL assays (fluorescence microscopy)**	TdT labels DNA strand breaks with tagged dUTPs (apoptosis-associated DNA fragmentation)	Preserves tissue architecture/3D context, combination with other dyes possible	Prior training/introduction in imaging is necessary, Embedding and sectioning required; multi-day protocol; long hands-on time	Fluorescence microscope, microtome/cryostat	€200 per 100 samples	2-3 days (≈6–8 h hands-on)	7	5	3	8

*Costs were calculated for consumables specific to this assay; standard cell-culture consumables (e.g., well plates, pipette tips) are not included.

**Seeding of cells/organoids and treatment periods, including incubations, are not included, analysis of obtained data is also not included; time varies with sample quantity (and imaging settings for image-based methods)

*** The lactate dehydrogenase assay is a similar approach.

Note:

Multi-well assays (e.g, SRB, ATP) are vulnerable to cell-number variability; with organoids, exact cell numbers are difficult to determine, organoids need dissociation for these assays (assays usually optimized for adherent 2D cells)

Numbers in the rigth part of the table indicate numerical ratings on a scale from zero to ten for sensitivity, specificity, throughput, and information content (0 = lowest, 10 = highest). Scores represent the median of independent ratings from multiple co-authors.

Practical integration

It is often advantageous to combine assays. In a drug-testing workflow, begin with high-throughput, lower-specificity plate assays (e.g., ATP/luminescence) to screen a broad panel, then validate promising compounds with flow cytometry and/or imaging to gain higher specificity and, where needed, single-cell or spatial information. This staged approach balances scale with mechanistic insight.
