# Supplementary figures and images for: Flow cytometry protocol for cell death analysis in glioblastoma organoids: A technical note
Source: PLoS One. 2025 Sep 23;20(9):e0327660. doi: 10.1371/journal.pone.0327660 (PMC12456761; doi:10.1371/journal.pone.0327660)

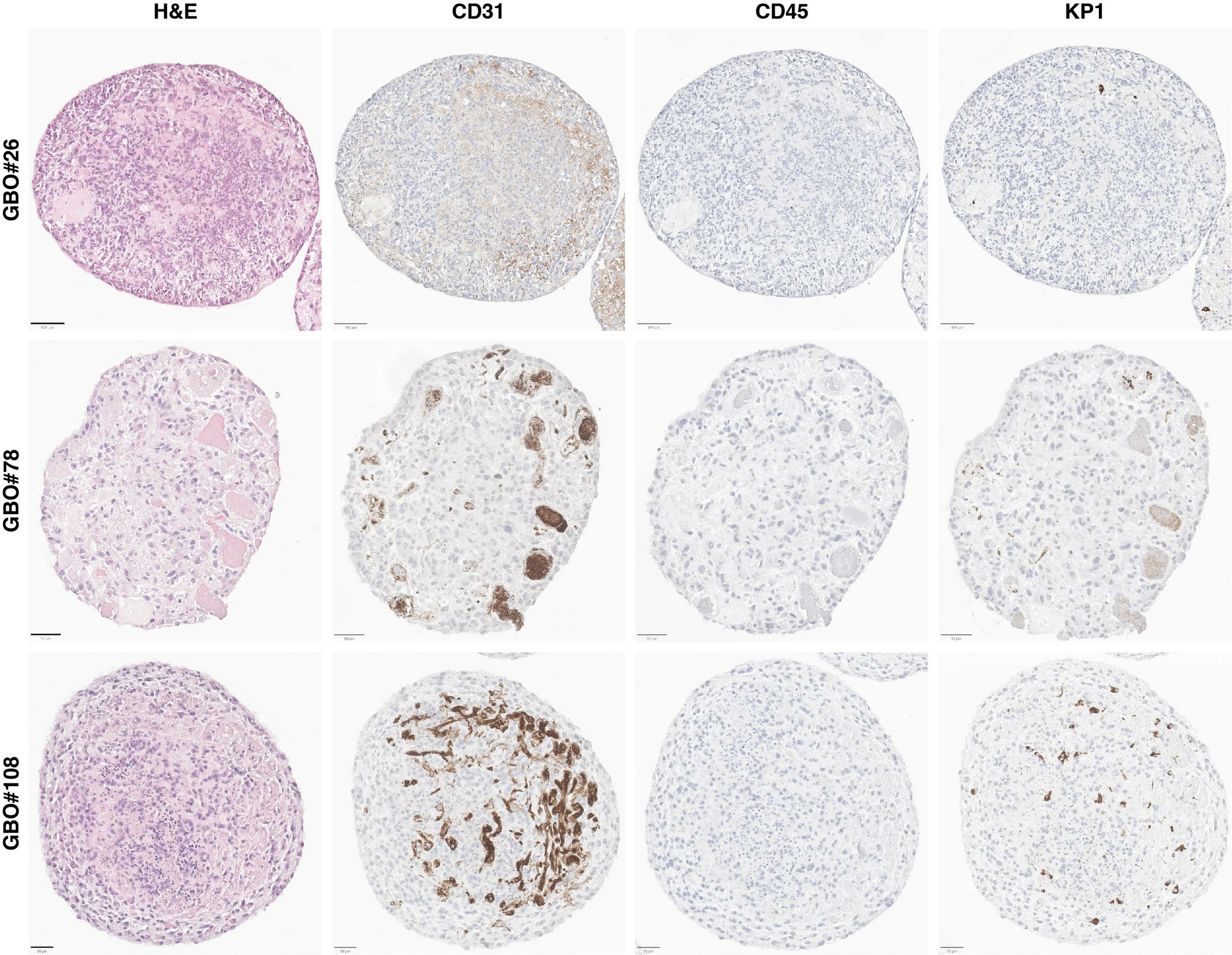

Supplement: S1 Fig — Immunohistochemical staining was performed on GBO#26, GBO#78, and GBO#108 using hematoxylin and eosin (H&E), CD31 (marker for endothelial cells and macrophages), CD45 (marker for leukocytes), and KP1 (marker for macrophages and activated microglia). Abbreviations: GBO, glioblastoma organoids. (TIF) [file pone.0327660.s005.tif]

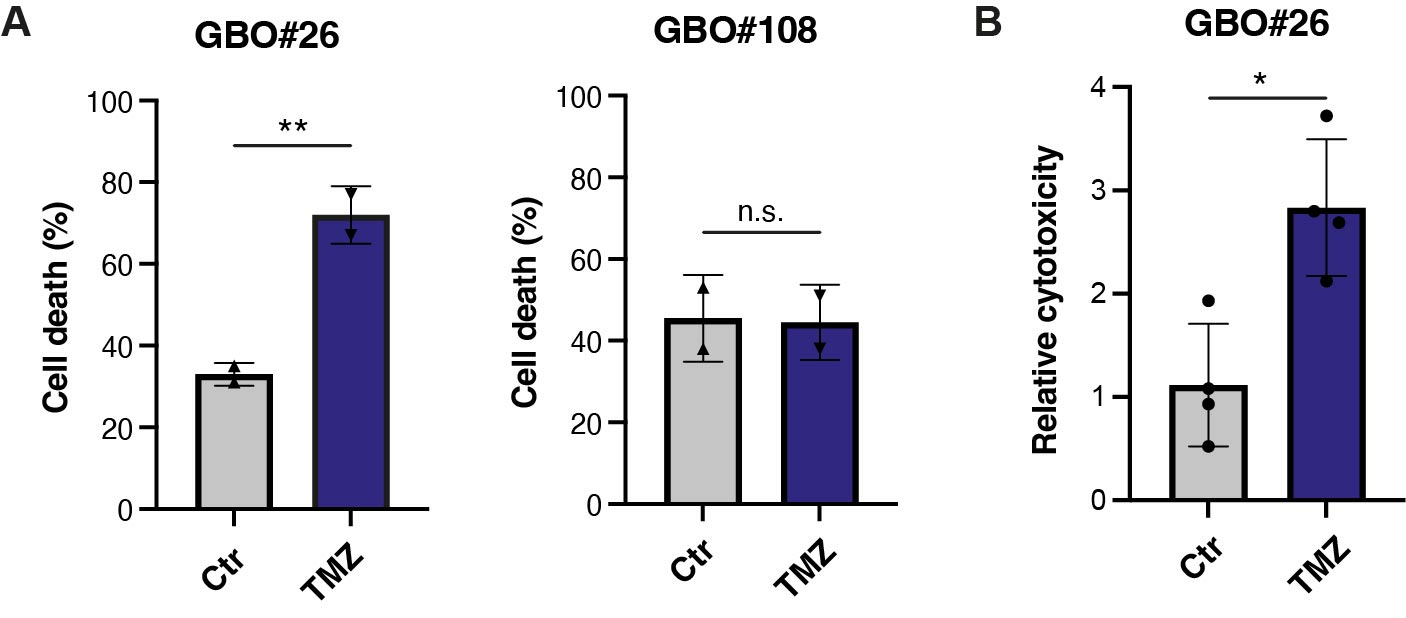

Supplement: S2 Fig — (A) Barplot showing cell death rates from flow cytometry experiments based on Hoechst staining in GBO#26 and GBO#108. (B) Relative cytotoxicity values from the LDH assay for GBO#26 following TMZ treatment. * and ** denote p < 0.05 and p < 0.01, respectively. Abbreviations: Ctr, control; GBO, glioblastoma organoid; TMZ, temozolomide. (TIF) [file pone.0327660.s006.tif]
